# Rest

**Published:** 1871-10

**Authors:** 


### REST.

The mind is best rested by diversion to a different subject of thought, or by muscular activities; the most complete rest is that which sleep gives; after great mental excitement, sleep is often impossible, but if there is diversion in muscular occupation for a while, refreshing sleep will follow.

Rachel, the greatest tragic actor of her day, on returning to her rooms after a theatrical performance of an extraordinarily exciting character, found most rest in changing the furniture of the room, or from one room to another; this had the double effect of diverting the blood from the brain, and of giving exit to the nervous excitement through the muscles.

Some persons, at the end of a public address, find themselves in such a state of mental excitement that they cannot rest, especially as the mind will run back on the performance, going round in a circle all the time. Such should go at once into lively company, or engage in active exercise or exciting work; if these are not practicable, the perusal of a newspaper or book of short sentences or proverbs is a good substitute.

If from any cause a man feels himself almost exhausted mentally or muscularly, a cup of hot, black tea affords an instantaneous and most delightful relief.

If very tired, physically, lie on the back, knees drawn up, the hands clasped above the head, or resting on the elbows, the fore-arm at right angles, and the hands hanging over by the bend of the wrists.

Some persons are best rested by lying on the face for a while. Sometimes persons become tired and restless in bed, being waked up, and cannot get to sleep again. Rest and sleep may be often had by getting up and using a

TOWEL BATH

thus: Take a towel, dip a corner of it in water, cold or warm, as is most agreeable, lay the dampened part flat on the hand, and with mouth shut and breast protruding rub the whole surface of the body, fast and hard, as far as can be reached in every direction, and then go to bed again; the feeling of refreshment and vigor, from such an operation, properly performed, is oftentimes most agreeable, to be followed by quick and delicious sleep, provided the mind is not bent on disagreeable topics. If so, it is better to get up, dress, and go to work, or take a trot on a horse, even if it be at midnight. These turmoils and wrestlings of the mind at night must be terrible.

